# LncRNA PLAC2 upregulates miR-663 to downregulate TGF-β1 and suppress bladder cancer cell migration and invasion

**DOI:** 10.1186/s12894-020-00663-w

**Published:** 2020-07-10

**Authors:** Zhenxing Zhang, Ping Ao, Hui Han, Qi Zhang, Yang Chen, Jie Han, Qunlian Huang, Houbao Huang, Dong Zhuo

**Affiliations:** Department of Urology, The first affiliated hospital of Wannan Medical College, No. 2, Zheshan West Road, Jinghu District, Wuhu City, Anhui Province 241001 P. R. China

**Keywords:** Bladder cancer, lncRNA PLAC2, miR-663, TGF-β1, Migration, Invasion

## Abstract

**Background:**

The roles of lncRNA PLAC2 in bladder cancer (BC) were explored.

**Methods:**

The expression of PLAC2 in two types of tissue of BC patients was detected by RT-qPCR and the expression data were compared by paired t test. The 56 patients were staged according to the AJCC criteria, and 12, 15, 15 and 14 cases were classified into stage I-IV, respectively. The expression of TGF-β1 and miR-663 in BC tissues were also detected by RT-qPCR experiments.

**Results:**

Our data showed that the expression levels of PLAC2 were significantly lower in BC tissues than that in non-cancer tissues. The expression of PLAC2 was not affect by clinical stages and low expression levels of PLAC2 predicted lower survival rate. The expression of PLAC2 was positively correlated with miR-663 and inversely correlated with TGF-β1 in BC tissues. In BC cells, downregulated TGF-β1 and upregulated miR-663 were observed after the overexpression of PLAC2. Overexpression of PLAC2 also resulted in suppressed invasion and migration of BC cells. Overexpression of miR-663 resulted in downregulated TGF-β1 but did not affect the expression of PLAC2. Overexpression of TGF-β1 reduced the inhibitory effects of overexpression of PLAC2 and miR-663 on cell migration and invasion.

**Conclusion:**

PLAC2 can upregulate miR-663 to downregulate TGF-β1 and suppress BC cell migration and invasion.

## Background

Incidence of bladder cancer (BC) is one of the most common types of urological malignancy [1]. BC affects about 400, 000 new cases annually and about 40% of these patients die of BC within 5 years after the initial diagnosis [2]. Treatment of bladder cancer, such as surgical resection, has been improved significantly during the past several decades. However, most patients are diagnosed at advanced stages, which are not appropriate for surgery [3, 4]. Systemic therapies and targeted therapies have shown promising potentials in the treatment of BC [5], while effective targets are limited and novel targets are urgently needed.

Long (> 200 nt) non-coding RNAs (lncRNAs) participate in human cancer biology by regulating the expression of tumor suppressive and oncogenic pathways [6, 7]. Almost all the classic cancer-related pathways, such as the TGF-β signaling, have crosstalk with lncRNAs [8, 9]. It is generally believed that regulation of lncRNA expression may contribute to cancer treatment, while the functions of most lncRNAs are barely known [10]. LncRNA PLAC2 was demonstrated to be a cell cycle inhibitor in glioma, indicating its role as a tumor suppressor [11]. MiR-663 in cancer biology can target TGF-β1 to inhibit cancer development [12], while TGF-β1 can interact with the STAT signaling [13], which is regulated by PLAC2 [11]. The present study was carried out to explore the role of PLAC2 in BC with a focus on its interactions with miR-663 and TGF-β1.

## Methods

### Patient admission and follow-up

The present study enrolled 56 BC patients from 166 BC patients admitted to the first affiliated hospital of Wannan Medical College between July 2010 and December 2013. Inclusion criteria were:1) newly diagnosed cases; 2) patients were willing to participate in the 5-year follow-up and completed follow-up. Exclusion criteria were: 1) recurrent BC; 2) other clinical disorders were observed; 3) any therapies were initiated within 3 months before admission; 4) previous or family history of malignancies; 4) patients lost or died of other causes during follow-up. The 56 patients were staged according to the AJCC criteria, and 12, 15, 15 and 14 cases were classified into stage I-IV, respectively. Based on the criteria of urothelial carcinoma grading established by American College of Surgeons, the 56 patients included 26 cases with high grade and 30 cases with low grade. This study was approved by the Ethics committee of aforementioned hospital. All patients were informed of the details of experiments and possible publication of this study, and they all signed the informed consent.

### Tissues

All patients were diagnosed by histopathological biopsy. During biopsy, BC and adjacent (within 3 cm around tumors) non-cancer tissues (0.08–0.12 g) were collected from 3 different positions of the tumor. All tissues were confirmed by histopathological examinations.

### Cells and transient transfections

Human BC cell line HT-1197 (ATCC, USA) was used. Eagle’s Minimum Essential Medium was mixed with 10% fetal bovine serum (FBS) and was used as the culture medium. Cells were cultivated at 37 °C with 5% CO_2_. PLAC2 and TGF-β1 expression vectors were constructed using pcDNA3.1 vector (Sangon, Shanghai, China). Negative control miRNA and miR-663 mimic were purchased from Sigma-Aldrich (USA). Inhibitor negative control and miR-663 inhibitor were also purchased from Sigma-Aldrich (USA). HT-1197 cells were harvested at the confluence of 70–90%. Next, 10 nM of LAC2 and TGF-β1 expression vector, 10 nM of empty pcDNA3.1 vector (negative control, NC), 30 nM of negative control (NC) miRNA and miR-663 mimic, or 30 nM of inhibitor negative control (NC) and miR-663 inhibitor were transfected into 10^5^ cells using lipofectamine 2000 transfection reagent (Sigma-Aldrich, USA). Cells without transfections were used as the control (C). the following experiments were performed using cells collected at 24 h post-transfection.

### RT-qPCR

HT-1197 cells as well as tissues (ground in liquid nitrogen before use) were mixed with VWR Life Science RiboZol™ RNA Extraction Reagent (VWR, USA) to extract total RNAs. Following DNase I digestion to remove genomic DNA, AMV Reverse Transcriptase (Canvax Biotech, USA) was used to synthesize cDNA through reverse transcription. The qPCR mixtures were prepared using KAPA SYBR FAST qPCR Master Mix (Kapa Biosystems). The expression of PLAC2 and TGF-β1 were determined with GAPDH as the endogenous control. HT-1197 cells as well as tissues were also used to extract miRNAs using mirVana miRNA Isolation Kit (Thermo Fisher Scientific). Following miRNA reverse transcriptions using MystiCq® microRNA cDNA Synthesis Mix (Sigma-Aldrich, USA), all qPCR reaction mixtures were prepared using miScript SYBR Green PCR Kit (QIAGEN, Germany). The expression of miR-663 was analyzed with U6 as endogenous control. Three biological replicates were included in each experiment and data were processed using 2^-ΔΔCT^ method. Primer sequences were: TGF-β1: (forward) 5′-TACCATGCCAACTTCTGTCTGGGA-3′ and (reverse) 5′-ATGTTGGACAACTGCTCCACCTTG-3′; PLAC2 (forward) 5′-AATGTCTTGGCCTTGAATGA-3′ and (reverse) 5′-CAAACTCAGGGATACATGGA-3′; GAPDH (forward) 5′-GCACCGTCAAGGCTGAGAAC-3′ and (reverse) 5′-TGGTGAAGACGCCAGTGGA-3′; miR-663: (forward) 5′-AGGCGGGGCGCCGCGGGACCGC-3′. U6 primers and miR-663 reverse primers were included in the kit.

### Cell migration and invasion assay

HT-1197 cells (3 × 10^4^) were harvested at 24 h after transfections and mixed with 1 ml serum-free Eagle’s Minimum Essential Medium to prepare single cell suspensions. Cells were transferred to the upper Transwell chamber (0.1 ml per well) and the lower chamber was filled with Eagle’s Minimum Essential Medium (20% FBS). The membrane was coated with Matrigel (356,234, Millipore, USA) before invasion assay to mimic invasion condition. In migrtaion assay, uncoated membranes were used. Cells were cultivated at 37 °C with 5% CO_2_ for 8 h, and membranes were cleaned and stained with 1% crystal violet (Sigma-Aldrich, USA) at 22 °C for 15 min. Stained cells were observed under an optical microscope.

### Western blot

HT-1197 cells (3 × 10^5^) were harvested at 24 h after transfections and mixed with 1 ml RIPA solution (Beyotime, Jiangsu, China) to extract total proteins. Proteins were denatured and subjected to 12% SDS-PAGE gel electrophoresis. Membranes were first incubated with 5% non-fat milk at 22 °C for 2 h, followed by incubation with TGF-β1 and GAPDH rabbit polyclonal primary antibodies at 4 °C overnight. Then membranes were incubated with IgG-HRP goat anti rabbit (1:1000, MBS435036, MyBioSource) secondary antibody at 22 °C for 2 h. Signals were developed using ECL (Sigma-Aldrich, USA) and Image J v1.46 software was used to process data.

### Statistical analyses

Means values presented in this study were calculated using the data from 3 biological replicates. Differences between two types of tissue were compared using paired t test. ANOVA Tukey’s test was used to compare differences among different cell and patient groups. Correlations were analyzed using linear regression. The 56 BC patients were divided into high (*n* = 26) and low (*n* = 30) PLAC2 level groups (Youden’s index). Survival curves were plotted using K-M method and compared by log-rank test. Chi-squared test was performed to analyze the correlations between the expression levels of PLAC2 and patients’ clinical data. *P* < 0.05 was statistically significant.

## Results

### PLAC2 were significantly downregulated in BC

**Fig. 1:**
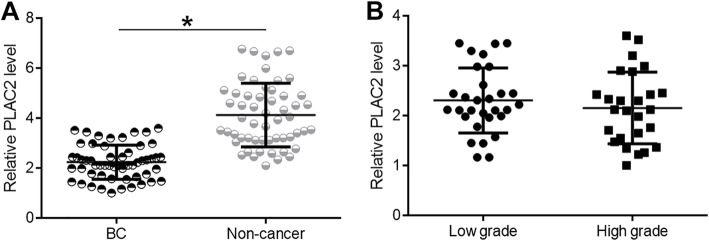
PLAC2 were significantly downregulated in BC. PLAC2 detected by RT-qPCR and analyzed by paired t test showed that expression levels of PLAC2 were significantly lower in BC tissues compared to that in non-cancer tissues (**a**). Expression levels of PLAC2 were slightly lower in high grade group than in low grade group, but the difference was not significant (**b**). (*, *p* < 0.05)

The expression of PLAC2 in two types of tissue of BC patients (*n* = 56) were detected by RT-qPCR and the expression data were compared by paired t test. The data revealed that the expression levels of PLAC2 were significantly lower in BC tissues in comparison to that in non-cancer tissues (Fig. 1a *p* < 0.05). The expression levels of PLAC2 were slightly lower in high grade group than that in low grade group, but the difference was not significant (Fig. 1b, *p* < 0.05). Chi-squared test showed that the expression levels of PLAC2 were not significantly correlated with patients’ age, gender, multifocal tumors, CIS, cancer stages and tumor grades (Table 1).

**Table 1:** Correlations between the expression levels of PLAC2 and patients’ clinical data.

Variables	Groups	Cases	High (*n* = 26)	Low (*n* = 30)	χ^2^	*p* value
Age	> 65 (years)	34	16	18	0.01	0.91
	<= 65 (years)	22	10	12		
Gender	Male	40	19	21	0.06	0.80
	Female	16	7	9		
Multifocal tumors	Yes	24	10	14	0.38	0.64
	No	32	16	16		
CIS	Yes	12	5	7	0.14	0.71
	No	44	21	23		
Tumor stage	I	12	5	7	0.47	0.93
	II	15	7	8		
	III	15	8	7		
	IV	14	6	8		
Tumor grade	High	26	12	14	0.01	0.97
	Low	30	14	16		

### PLAC2 was not affect by clinical stages and low expression levels of PLAC2 predicted lower survival rate

**Fig. 2:**
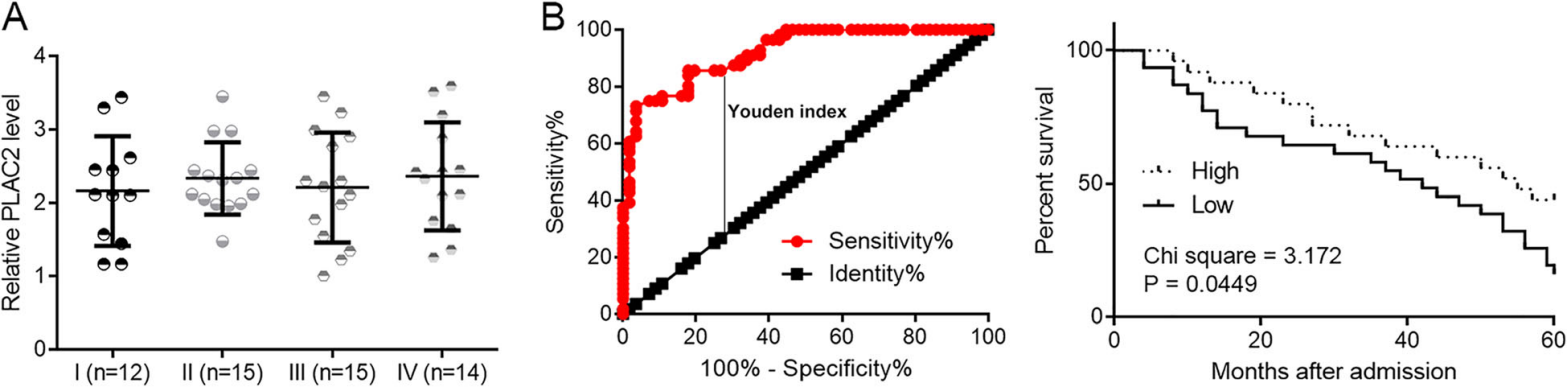
PLAC2 was not affect by clinical stages and low PLAC2 levels predicted low survival rate. One-way ANOVA and Tukey test analysis showed no significant differences in expression levels of PLAC2 in BC tissues among different clinical stages (**a**). Survival curve analysis showed that patients with low levels of PLAC2 had significantly worse overall survival rate (**b**)

The 56 patients were staged according to the AJCC criteria, and 12, 15, 15 and 14 cases were classified into stage I-IV, respectively. The expression levels of PLAC2 in BC were compared among different clinical stages using one-way ANOVA and Tukey test. No significant differences were observed among different clinical stages (Fig. 2a). In addition, survival curves showed that patients with low expression levels of PLAC2 had significantly worse overall survival rate (Fig. 2b). It is worth noting that the 56 patients included 26 cases with high grade and 30 cases with low grade. The expression levels of PLAC2 were slightly lower in high grade group in comparison to that in the low-grade group, but the difference was not significant (*p* < 0.05, data not shown).

### PLAC2 was positively correlated with miR-663 and inversely correlated with TGF-β1 in BC tissues

**Fig. 3:**
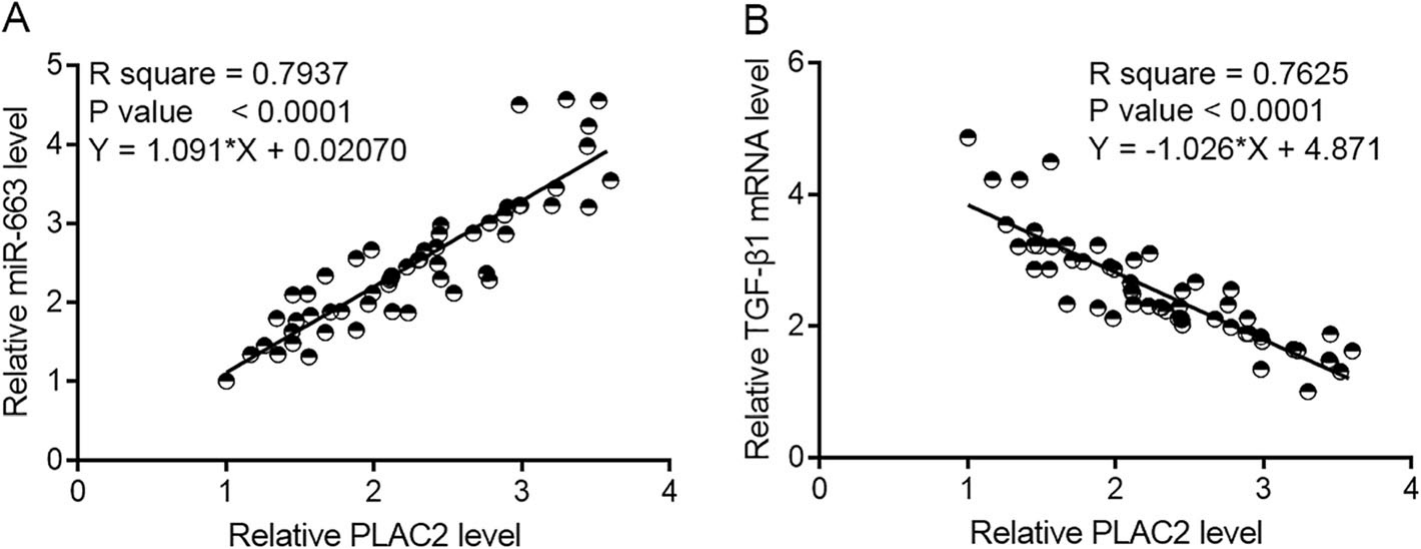
PLAC2 was positively correlated with miR-663 and inversely correlated with TGF-β1 in BC tissues. Linear regression showed that PLAC2 was positively correlated with miR-663 (**a**) and inversely correlated with TGF-β1 (**b**) in BC tissues

The expression of TGF-β1 and miR-663 in BC tissues were also detected by RT-qPCR experiments. Correlations were analyzed by linear regression. It was observed that the expression of PLAC2 was positively correlated with the expression of miR-663 (Fig. 3a) and inversely correlated with TGF-β1 (Fig. 3b) across BC tissues.

### A novel PLAC2/miR-663/TGF-β1 pathway is involved in the regulation of BC cell migration and invasion

**Fig. 4:**
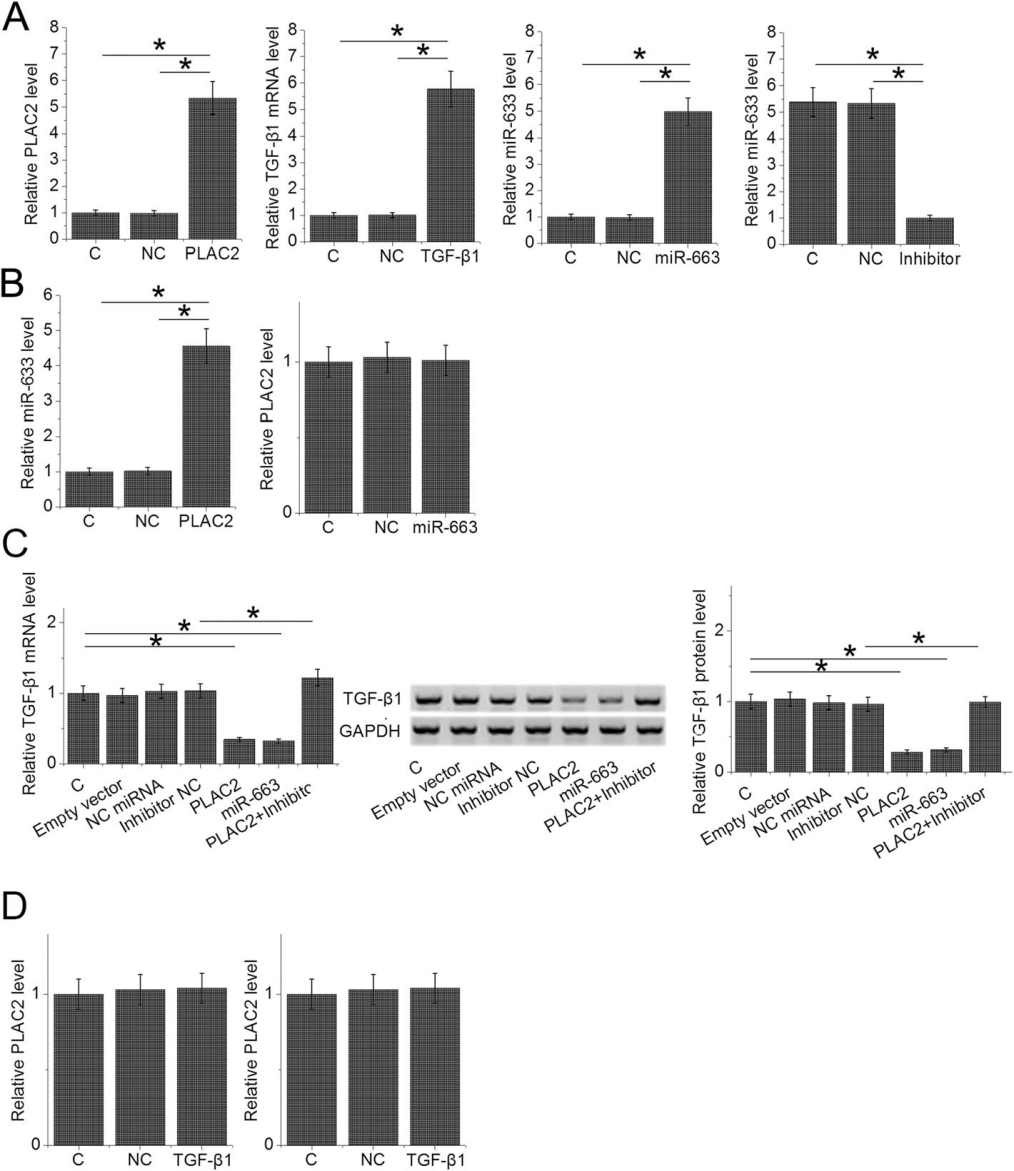
A novel PLAC2/miR-663/TGF-β1 pathway was characterized. RT-qPCR results showed the altered expression of PLAC2, miR-663 and TGF-β1 at 24 h after transfections (**a**). Overexpression of PLAC2 resulted in upregulation of miR-663, while overexpression of miR-663 resulted in no significantly changed expression levels of PLAC2 (**b**). In addition, overexpression of PLAC2 and miR-663 resulted in the downregulation of TGF-β1, while miR-663 inhibitor attenuated the effects of PLAC2 overexpression (**c**). Moreover, overexpression of TGF-β1 did not significantly affect the expression of PLAC2 and miR-663 (**d**) (*, *p* < 0.05)

**Fig. 5:**
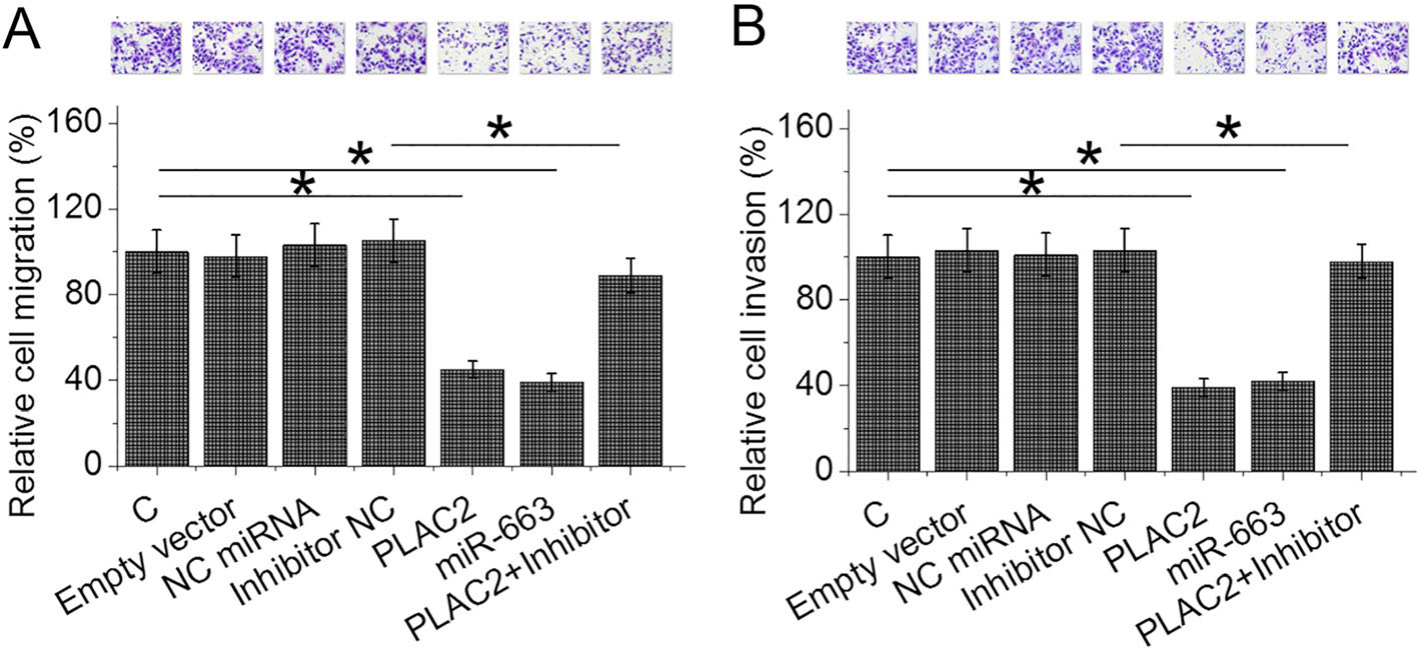
PLAC2/miR-663/TGF-β1 pathway is involved in the regulation of BC cell migration and invasion. Overexpression of PLAC2 and miR-663 resulted in reduced rates of BC cell migration (**a**) and invasion (**b**), while overexpression of TGF-β1 resulted in the increased rates. In addition, miR-663 inhibitor reduced the effects of PLAC2 overexpression (*, *p* < 0.05)

After transfections, the expression levels of PLAC2, miR-663 and TGF-β1 were significantly altered at 24 h after transfections (Fig. 4a, *p* < 0.05), indicating the successful transfections. In was observed that overexpression of PLAC2 resulted in upregulation of miR-663, while overexpression of miR-663 showed no significantly changed expression levels of PLAC2 (Fig. 4b, *p* < 0.05). In addition, overexpression of PLAC2 and miR-663 resulted in the downregulation of TGF-β1, while miR-663 inhibitor attenuated the effects of overexpression of PLAC2 (Fig. 4c, *p* < 0.05). Moreover, overexpression of TGF-β1 did not significantly affect the expression of PLAC2 and miR-663 (Fig. 4d). Therefore, a novel PLAC2/miR-663/TGF-β1 pathway was characterized. It is worth noting that the targeting of TGF-β1 by miR-663 has been well established [12]. Transwell migration and invasion assays were performed to investigate the involvement of the PLAC2/miR-663/TGF-β1 pathway in regulating BC cell behaviors. It showed that overexpression of PLAC2 and miR-663 resulted in reduced rates of BC cell migration (Fig. 5a) and invasion (Fig. 5b), while overexpression of TGF-β1 resulted in increased rates (*p* < 0.05). In addition, miR-663 inhibitor reduced the effects of overexpressing PLAC2 (*p* < 0.05). It is worth noting that our preliminary CCK-8 assay resulted showed that PLAC2 had no significant effects on cell proliferation, apoptosis and stemness (data not shown).

## Discussions

The functions of PLAC2 in BC and its clinical potentials for BC have been investigated in this study. We found that PLAC2 was downregulated in BC and played a tumor suppressive role by downregulating TGF-β1 through the upregulation of miR-663, which can directly target TGF-β1 in glioblastoma [12].

The TGF-β signaling is a well-studied pathway in cancer biology [13]. TGF-β inhibits cancer cell proliferation and function as a tumor suppressor in most types of cancer at initiation stages [14], while it also promotes tumor metastasis at late stages [14]. Our study didn’t test the effects of overexpressing TGF-β1 on BC cell migration and invasion because it has been well established that TGF-β1 promotes the BC cell migration and invasion through the interactions with multiple downstream pathways [15]. However, the TGF-β signaling can also be inhibited by certain factors, such as miRNAs. A recent study reported that miR-663 can target TGF-β1 to inhibit the behaviors of glioblastoma cells [12]. Our study observed the downregulation of TGF-β1 in BC cell after the overexpression of miR-663. Therefore, miR-663 may also target TGF-β1 in BC cells. These data suggest that, although BC and glioblastoma are two types of cancer that affect different organs, they may share similar molecular pathogenesis [16, 17].

Cancer prognosis is always important because it not only predicts the survival of patients within a time period but also helps the development of individualized treatment. PLAC2 is found to be a tumor suppressor in glioma [11]. The expression data of PLAC2 are not available in TCGA and CCLE database and we investigated its expression and functionality in this study. We found that PLAC2 is also a tumor suppressor in BC. Different from the roles of PLAC2 as a cell cycle inhibitor in glioma, PLAC2 functions as a migration and invasion inhibitor in BC. Therefore, the same lncRNA may play different roles in different types of cancer, which highlights the complicated molecular pathogenesis of cancers. BC patients with low expression levels of PLAC2 showed low 5-year survival rate, indicating its prognostic value for BC. Interestingly, PLAC2 was found to be an upstream activator of miR-663 in BC cells, and the upregulation of miR-663 is accompanied by the downregulation of TGF-β1. Therefore, a novel PLAC2/miR-663/TGF-β1 pathway was characterized as a pathway involved in the regulation of BC cell behaviors. LncRNAs can inhibit miRNAs by serving as their endogenous sponge [18–20]. However, we observed the upregulation of miR-663 by PLAC2. We speculate certain mediators may exist between PLAC2 and miR-663. Our future studies will test this possibility.

Interestingly, the expression of PLAC2 was not significantly affected by clinical stages, while PLAC2 showed significant effects on cancer cell invasion and migration. It is well-known that different genetic factors participate in different stages of cancer [21]. We speculate that certain factors can inhibit the roles of PLAC2 in cell invasion and migration at early cancer stages, while it does not inhibit at advanced stages and in cancer cell lines. It is worth noting that we did not perform wound healing assay to measure the migration of cells under different conditions. Our future studies will perform wound healing assay to further confirm our conclusions. The present study only included one cell line. Our future studies will include more cell lines to further confirm our conclusions. The overexpression of TGF-β1 is only confirmed at mRNA level, which is a limitation. Our future study will solve this problem.

## Conclusions

In conclusion, PLAC2 was downregulated in BC and PLAC2 may upregulate miR-663 to downregulate TGF-β1, thereby inhibiting BC.

## Supplementary information

**Additional file 1.**

## Data Availability

The analyzed data sets generated during the study are available from the corresponding author on reasonable request.

## Abbreviations

BC: Bladder cancer
lncRNAs: Long (> 200 nt) non-coding RNAs
