# Supplementary figures and images for: LncRNA PLAC2 upregulates miR-663 to downregulate TGF-β1 and suppress bladder cancer cell migration and invasion
Source: BMC Urol. 2020 Jul 10;20:94. doi: 10.1186/s12894-020-00663-w (PMC7350696; doi:10.1186/s12894-020-00663-w)

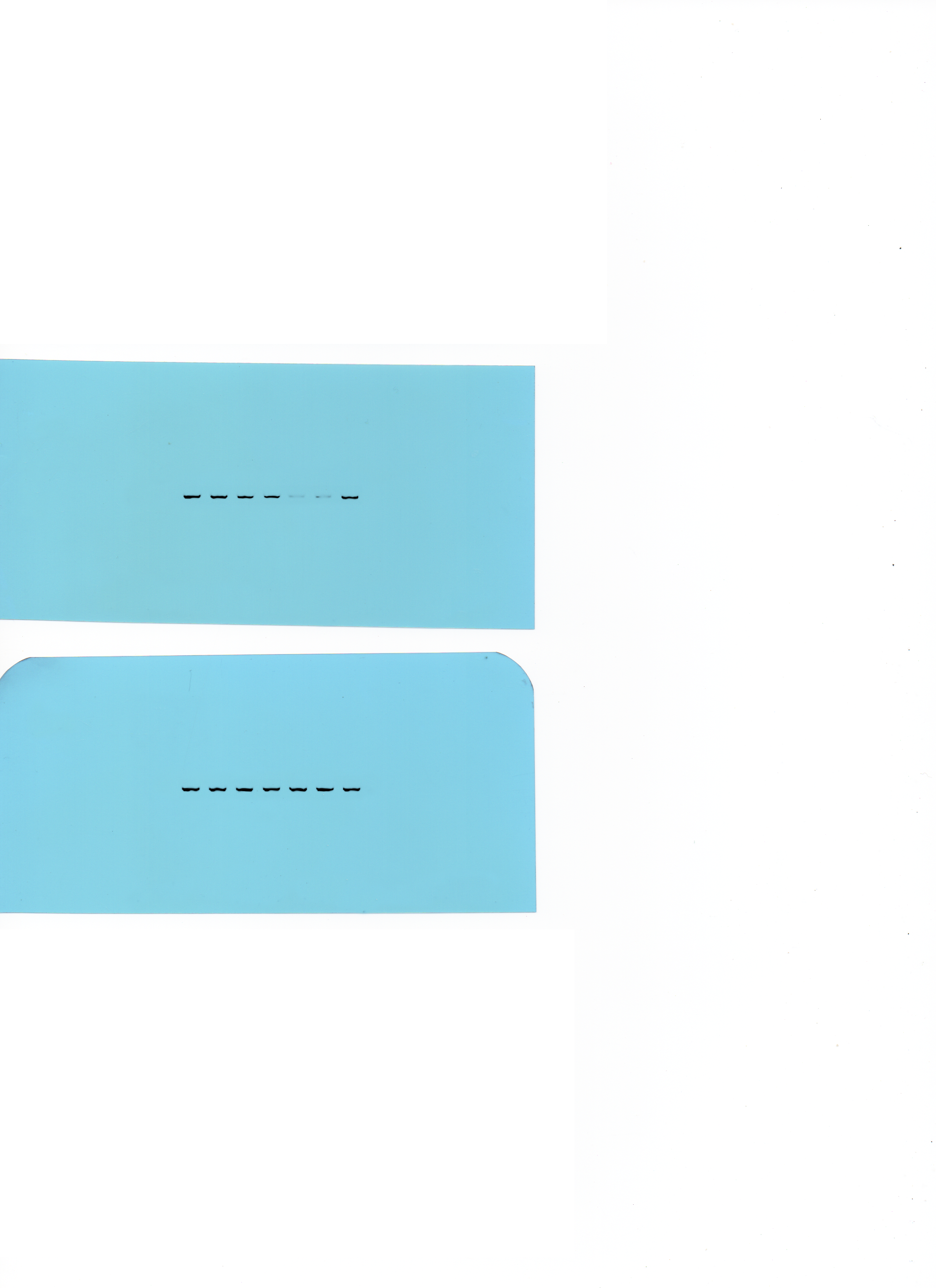

Supplement: Supplementary file 1 — Additional file 1. [file 12894_2020_663_MOESM1_ESM.tif]
